# The Role of Different Retinal Imaging Modalities in Predicting Progression of Diabetic Retinopathy: A Survey

**DOI:** 10.3390/s22093490

**Published:** 2022-05-04

**Authors:** Mohamed Elsharkawy, Mostafa Elrazzaz, Ahmed Sharafeldeen, Marah Alhalabi, Fahmi Khalifa, Ahmed Soliman, Ahmed Elnakib, Ali Mahmoud, Mohammed Ghazal, Eman El-Daydamony, Ahmed Atwan, Harpal Singh Sandhu, Ayman El-Baz

**Affiliations:** 1 Bioengineering Department, University of Louisville, Louisville, KY 40292, USA; mohamed.elsharkawy@louisville.edu (M.E.); mgelra01@louisville.edu (M.E.); a.sharafeldeen@louisville.edu (A.S.); fakhal01@louisville.edu (F.K.); ahmed.soliman@louisville.edu (A.S.); aaelna02@louisville.edu (A.E.); ahmahm01@louisville.edu (A.M.); harpal.sandhu@louisville.edu (H.S.S.); 2 Electrical, Computer and Biomedical Engineering Department, College of Engineering, Abu Dhabi University, Abu Dhabi 59911, United Arab Emirates; mohammed.ghazal@adu.ac.ae (M.G.); marah.alhalabi@adu.ac.ae (M.A.); 3 Information Technology Department, Faculty of Computers and Information, Mansoura University, Mansoura 35516, Egypt; emane_daydamoni@mans.edu.eg (E.E.-D.); ahmed.atwan@nbu.edu.sa (A.A.)

**Keywords:** diabetic retinopathy (DR), computer-aided diagnostic system (CAD), machine learning (ML), deep learning (DL), optical coherence tomography (OCT), OCT angiography (OCTA), fundus photography (FP)

## Abstract

Diabetic retinopathy (DR) is a devastating condition caused by progressive changes in the retinal microvasculature. It is a leading cause of retinal blindness in people with diabetes. Long periods of uncontrolled blood sugar levels result in endothelial damage, leading to macular edema, altered retinal permeability, retinal ischemia, and neovascularization. In order to facilitate rapid screening and diagnosing, as well as grading of DR, different retinal modalities are utilized. Typically, a computer-aided diagnostic system (CAD) uses retinal images to aid the ophthalmologists in the diagnosis process. These CAD systems use a combination of machine learning (ML) models (e.g., deep learning (DL) approaches) to speed up the diagnosis and grading of DR. In this way, this survey provides a comprehensive overview of different imaging modalities used with ML/DL approaches in the DR diagnosis process. The four imaging modalities that we focused on are fluorescein angiography, fundus photographs, optical coherence tomography (OCT), and OCT angiography (OCTA). In addition, we discuss limitations of the literature that utilizes such modalities for DR diagnosis. In addition, we introduce research gaps and provide suggested solutions for the researchers to resolve. Lastly, we provide a thorough discussion about the challenges and future directions of the current state-of-the-art DL/ML approaches. We also elaborate on how integrating different imaging modalities with the clinical information and demographic data will lead to promising results for the scientists when diagnosing and grading DR. As a result of this article’s comparative analysis and discussion, it remains necessary to use DL methods over existing ML models to detect DR in multiple modalities.

## 1. Introduction

Diabetes mellitus affects millions of adults all over the world. It increases the risk for death and devastating complications related to end-organ damage from the disease. It may lead to nephropathy, neuropathy, retinopathy, and many other diseases such as dementia, non-alcoholic steatohepatitis, psoriasis, metabolic syndrome, cardiovascular disease, and cancer. Most of the diabetes-related complications are caused by overlapping pathophysiology [1,2].

Diabetic retinopathy (DR) is a common diabetic complication, which is the main cause of retinal blindness in the US [3]. DR is a potentially devastating, vision-threatening condition which is considered as an inflammatory, neurovascular complication. It is also associated with microvascular damage, preceded by neuronal injury/dysfunction preceding clinical microvascular damage. Clinical research has demonstrated the factors that predict the development of retinopathy in diabetic patients. The main predictors of retinopathy progression are duration of diabetes and hemoglobin A1c. According to The Diabetes Control and Complications Trial (DCCT), these factors explain 11% of the risk of developing retinopathy [4]. Similarly, the Wisconsin Epidemiologic Study of DR (WESDR), a large population-based study, studied the effect of hemoglobin A1c, cholesterol, and blood pressure. It was found that they all may contribute to retinopathy. The effect of these factors is not enough to explain the risk of progression of the DR. Therefore, many other factors may play a role in the development of DR [5].

Strong genetic factors have been studied in multiple family studies. These have been suggested as one of the factors affecting DR development in both DM Type 1 and Type 2 [6]. Other biochemical pathways are linked to complication development. Other studies have proposed that several biochemical pathways, such as oxidative stress and activation of protein kinase, are linked to hyperglycemia and microvascular complications. These pathological processes are affecting the disease process through effects on signaling, cellular metabolism, and growth factors [7,8,9]. To understand DR pathology, it is important to view it as a shared pathophysiologic process that damages the pancreatic beta-cell. In addition, the same pathophysiological process may cause cell and tissue damage, leading to organ dysfunction. Understanding the common pathophysiology is key to providing a broad range of treatment options for this common and critical complication [10].

It is also highlighted that it is vital to develop more precise and timely methods for detecting the early stages of the disease and predict its course of progression. This is important as it allows techniques that can discover any change in the retinal structure event before any signs or symptoms evolve clinically [10]. With the recent advances in the imaging modalities, clinicians have been using retinal imaging as a major component in grading and diagnosis of DR. Different imaging modalities have been used in the diagnosis and screening of DR. These include fluorescein angiography (FA), optical coherence tomography (OCT), fundus photographs (FP), and OCT angiography (OCTA) [11]. These imaging techniques provide large numbers of detailed images of the retina, which allows detection of small changes with high resolution level. However, the abundantly available images are hard to manually analyze during clinical practice. Moreover, the data related to retinal diseases are affected by increasing age. Therefore, the imaging data may change with the rising life expectancy.

Many earlier reviews have provided an extensive study of comprehensive retina assessment components and correlation with the levels of DR, as well as management for DR [12,13,14,15]. The current review discusses the classification of DR severity, and the role of the different imaging modalities in the management of the DR.

## 2. Clinical Staging of Diabetic Retinopathy Using Retinal Imaging

Evaluation of DR includes much information that raises the importance of a structured and well-studied framework to standardize terminology and the sharing of data among the healthcare providers who play a role in the management of diabetic patients. Therefore, simplified clinical disease severity scales were developed. The Early Treatment DR Study (ETDRS) and the WESDR are now the cornerstones for the clinical classification systems used internationally [16,17]. Both of these classifications have studied DR and diabetic macular edema (DME), focusing on the risk of progression and correlating each stage to certain level of risk. In addition, this assessment was very helpful in risk stratification and staging the DR findings in various clinical settings and accordingly led to evidence-based clinical recommendations for DR management.

**Figure 1:**
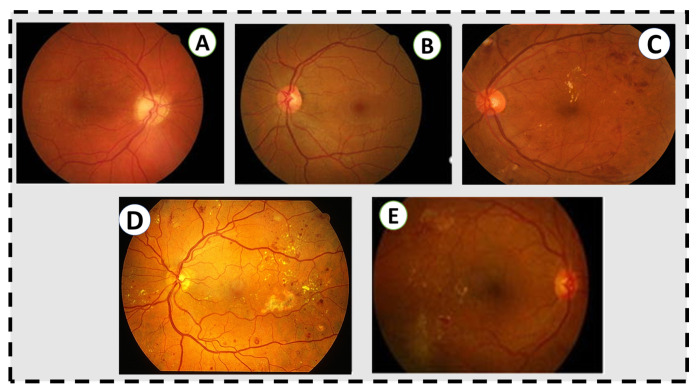
Grading in the fundus image. (**A**) No retinal disease. (**B**) Mild NPDR. (**C**) Moderate NPDR. There are some microaneurysms, dot and blot hemorrhages in the temporal macula, and a few flecks of lipid exudate, but no venous beading or other microvascular abnormalities. (**D**) Severe NPDR. There are abundant microaneurysms, dot and blot hemorrhages, extensive lipid exudates, and intraretinal microvascular abnormalities. (**E**) Active proliferative diabetic retinopathy, untreated, with neovascularization at the arcades and intraretinal lipid exudates and hemorrhages in the temporal macula.

**Figure 2:**
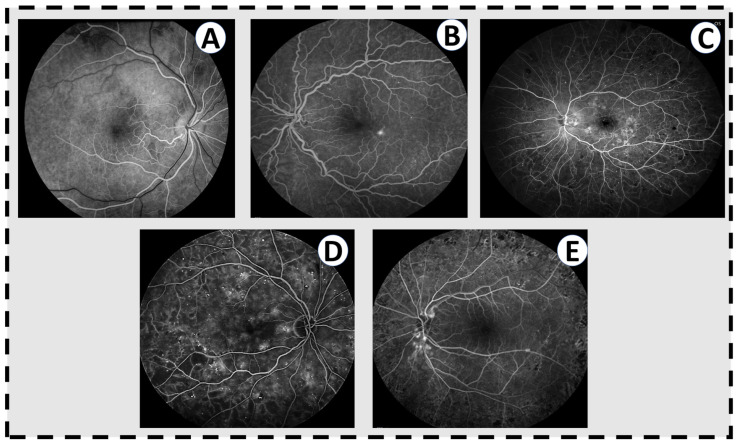
Fluorescien angiograms for varying levels of DR. (**A**) A normal angiogram in the early phase, where the arteries have filled with fluorescein dye but the veins have not. There is nothing in this eye. (**B**) Mild nonproliferative DR with a few scattered microaneuryms and a single pinpoint area of leakage inferotemporal to the fovea. (**C**) Moderate nonproliferative DR with multiple microaneurysms throughout the fundus, significant leakage of dye throughout the macula, and blockage in the periphery from intraretinal hemorrhages. (**D**) Severe nonproliferative DR with abundant microaneurysms and dark areas on the angiogram corresponding to capillary non-perfusion. (**E**) Active proliferative DR with leakage from the optic disc from neovascularization. The retinopathy has been treated with laser (dark spots in the periphery) but there remains some level of neovascular activity.

ETDRS guidelines are currently considered the gold standard for staging DR. ETDRS employs data from color FP, intravenous fluorescein angiography (IVFA), and dilated fundus exam findings, and stratifies disease severity using quadrant analysis. Grading criteria consist of multiple findings. It includes cotton wool spots, exudates, microaneurysms, neovascularization, and retinal hemorrhages [18,19,20]. However, the complexity of the severity scale made it impractical in clinical settings. Therefore, the full ETDRS severity scale is not used by most physicians [21]. However, various other systems for grading DR are commonly employed, including the International Clinical DR (ICDR) scale, which is recognized in either clinical or CAD environments. For DME, there are four severity scores, while there are five for DR. This scale requires a smaller field of view for DR. We describe the ICDR levels below, which are also presented in Table 1. Figure 1 and Figure 2 show the normal retina against four severity levels of DR based on FP and FA images (mild NPDR, moderate NPDR, severe NPDR, and PDR).

**Table 1:** Characteristics of the DR stages.

Stage	Characteristic
Normal	No retinal disease.
Mild NPDR	This stage contains a microaneurysms which are a small amount of fluid in the retinal blood vessels, causing the macula to swell.
Moderate NPDR	Retinal blood vessels become blocked due to their increased swelling, prohibiting the retina from being nourished.
Severe NPDR	Larger areas of retinal blood vessels are blocked, sending signals to the body to generate new blood vessels in the retina.
PDR	New blood vessels are generated in the retina abnormally, often leading to fluid leakage due to their fragility, causing a reduced field of vision, blurring, or blindness.

IVFA is for primary assessment of the retinal vasculature. It makes it easy to identify variable vascular abnormalities such as neovascularization, capillary nonperfusion, and disruption of the blood–retinal barrier [22,23,24]. However, histological images are still better than IVFA to examine the lower capillary density values and retinal capillary networks [25]. In conclusion, IVFA has many limitations. It is time-consuming, invasive, and occasionally causes nausea, pruritus, and even anaphylaxis. In addition, it is limited in its resolution [26,27,28].

Eyes with DR are classified according to international classification in different DR severity stages; non-proliferative (NPDR) and proliferative (PDR) stages, with and without macular edema [5]. There are multiple classification systems; however, the most clinically important are those assessing the risk for disease progression and vision loss due to DR. There are five grades of retinopathy classified according to the risk of progression. Eyes with severe NPDR are considered to be at high risk for developing PDR. A more simple clinical approach has been developed for assessing the severity of the DR to avoid the complexity of ETDRS [29].

Reviewing of ETDRS proposed that the 4:2:1 rule should be used as the main simplified method of classification of severe NPDR. Severe NPDR may include eyes with quadrants that have two quadrants containing definite venous beading (VB), extensive retinal hemorrhages (approximately 20/quadrant), or any quadrant containing definite intraretinal microvascular abnormalities (IRMA). Around 17% of eyes with NPDR will develop the high-risk proliferative disease within one year, while 44% will do in 3 years. Difficulty in recognizing IRMA and VB is a major concern. That is why more surrogate markers such as microaneurysms or retinal hemorrhages have been evaluated to be used as a marker for severe NPDR, as they are easy to recognize. Reevaluation of data from the WESDR showed that IRMA and VB are still more reliable in the prediction of the risk of progression to PDR. Microaneurysms and retinal hemorrhages lack the concordance; therefore, microaneurysms and retinal hemorrhages alone are not enough to predict the risk of progression. Moreover, hemorrhages alone were strongly related to risk of progression of either IRMA or VB; however, they were not as strong in predicting the risk of progression to PDR.

Hard or soft exudates are not indicative of the presence of VB or IRMA. Therefore, specific identification of IRMA and VB is crucial and should not depend on exudates or H/MA alone to discriminate among moderate and severe NPDR. Therefore, a description of this clinical disease severity scheme should include photographs of IRMA and VB. Moderate NPDR has findings that are more than exclusively microaneurysm and more than the 4:2:1 rule.

The ICDR clinical disease severity scale is meant to be a practical and reliable method of grading the severity of DR and DME. This system is intended to allow all the healthcare providers dealing with DR to grade the severity of the disease. However, the implementation of this staging system is mainly affected by examiner skills and the available equipment. The more precise and appropriate the grading of the DR, the more effective the management of the cases and timely referrals to highly specialized treatment centers. It is important to know that this system is mainly for grading and not for the treatment of DR. It is recognized that a better staging system would be implemented in the treatment guidelines and protocols, similar to the ETDRS and DR study. However, the variable healthcare delivery systems, as well as specific practice pattern, may lead to different management recommendations. This staging system is mainly targeting experienced ophthalmologists and skilled healthcare teams. However, hopes are high to use this staging system to provide common knowledge that would allow a consistent means of communication between all healthcare providers dealing with patients with DR. Therefore, the success of this system relies mainly on the wide range of exposure to ophthalmologists, other retinal care providers, and all related specialties such as primary care physicians, endocrinologists, podiatrists, and diabetologists. All the healthcare providers dealing with patients with diabetes should be familiar with these scales. The common standards and evaluation structure would provide similar care in managing DR among different providers. Nonetheless, continuous review and evaluation of the usefulness and practicality of using this system should be implemented to adopt any changes that might be needed in certain cases or settings.

In April 2002, the Global DR Group developed a new severity scale for DR to avoid the disadvantages of using the ETDRS severity scale. ETDRS was not easy to use or practical because it may have levels higher than those required for clinical evaluation. Therefore, the assessment becomes more complicated and requires high levels of skills and experience. In addition, complicated data require a standard means of sharing information and common terminology, which was not available [30,31,32]. The new disease severity scale consists of five levels. These levels are arranged in an increasing manner according to the severity and the risk of progression of DR. If there is no apparent retinopathy, it is the first level. The second level is considered mild NPDR, and it includes ETDRS stage 20 (microaneurysms only). For the first and second levels, low risk for progression is expected.

ETDRS levels are included in moderate NPDR, which is considered the third level. In this level, there is a significant risk of progression of the disease. Severe NPDR, which is the fourth level, has the highest risk for progression to PDR. The fifth level is proliferative DR. High risk with significant rate of progression indicates the fifth level. All eyes in this level have vitreous hemorrhage or neovascularization. Differentiating the diseased eyes into eyes with or without DME is a critical initial step. DME is an important structural complication of DR and the most common cause of blindness in DR. However, it has not traditionally been used to grade the overall level of retinopathy, as it can occur at any level of retinopathy. Nonetheless, it is important for clinicians to identify DME because of its effects on vision and our ability to treat it effectively. Two features in these eyes may help to lessen the variations in the examiner’s education and availability. In the first level, lipid in the posterior pole or apparent retinal thickening should be evaluated. In the second level, details of the retinal thickening and lipid from the fovea should be documented. If there is foveal involvement, this eye is considered to show severe DME. The eyes then can be classified according to the distance between the lesion and the macula. While it is distant from the macula, this is considered mild DME. If it is close to the fovea, it is graded as moderate DME. This severity scale helps in appropriate management through proper grading of the severity, and accordingly leads to more consistent referrals to highly specialized treatment centers. The characteristics of DR stages are summarized in Table 1.

## 3. Imaging Modalities for Diabetic Retinopathy

Multiple imaging techniques have been used widely in ophthalmology evaluation. FA, OCT, OCTA, and FP imaging are recently widely implemented by the ophthalmologists. It is worth noting that the more data that are available, the harder it is becoming to analyze the data manually. Therefore, automated systems to analyze the huge amount of data have been developed. Ophthalmoscopy, both direct and indirect, has been used to evaluate the DR. In addition, colored FP, single-field photography, and FA have been widely used. In the next subsections, we provide individual overviews for each modality used in DR diagnosis/grading.

### 3.1. Fluorescein Angiography (FA)

FA has historically been an important imaging modality for the assessment of DR, and still remains so to this day. It was the gold standard for evaluating capillary non-perfusion, ischemia, and neovascularization (NV) in the retina. It is particularly sensitive for this last feature, identifying areas of NV that are not identified in clinical examinations. However, this modality is invasive, time-consuming, and unwieldy. It involves placing an intravenous line in a patient, infusing fluorescein, and taking multiple photos of the patient over 10–15 min. Patient cooperation must be high in order to take useful images, something that can be challenging in patients with multiple co-morbidities. Figure 2 shows FA images for varying levels of DR against healthy retina.

### 3.2. Optical Coherence Tomography (OCT)

**Figure 3:**
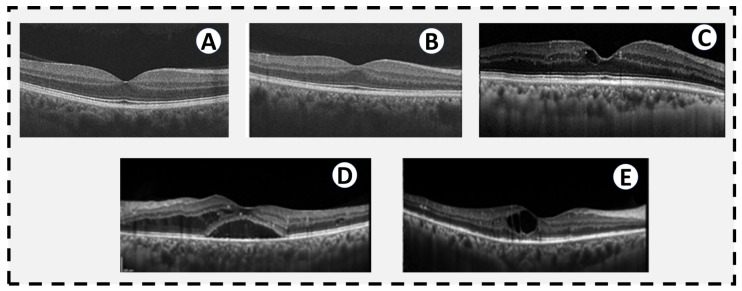
OCTs of different levels of diabetic retinopathy. (**A**) A normal OCT in a patient without diabetes. (**B**) OCT from a diabetic patient without DR by traditional historical criteria, but subtle changes in thickness and reflectivity of some of the retinal layers. (**C**) Mild NPDR with a small cystic space at the fovea. (**D**) Severe NPDR with diffuse diabetic macular edema extending into the subretinal space. (**E**) PDR with a large central cystic space and intraretinal hyperreflective spots temporally indicative of intraretinal lipid exudates. There is mild thinning of the temporal inner retina consistent with ischemia seen in PDR.

OCT is one of the most common imaging modalities used to evaluate DR. It projects a pair of near-infrared light beams into the eye to provide images of the retina. The reflected images are mainly affected by the thickness and the reflectivity of the retinal structures. The emerging beams are reflected on the measuring system [33]. OCT provides cross-sectional images of the retina and allows the measurement of the thickness of the retina. Cross-sectional images allow quantitative assessment of the thickness of the retina, which is crucial in the evaluation of the DME [34]. Figure 3 shows the different grades of DR against healthy retina.

### 3.3. Optical Coherence Tomography Angiography (OCTA)

**Figure 4:**
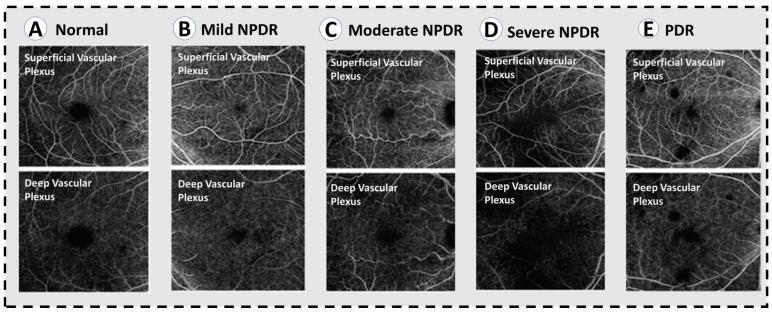
OCT angiograms across varying severities of diabetic retinopathy. (**A**) Normal OCTA. (**B**) Mild NPDR, showing mild loss of vessel density. (**C**) Moderate NPDR with lower vessel caliber and further loss of vessel density. (**D**) Severe NPDR, showing significant areas of capillary non-perfusion in the superifical and deep plexuses, as well as microaneurysms. (**E**) PDR, showing similar vascular changes to severe NPDR.

**Figure 5:**
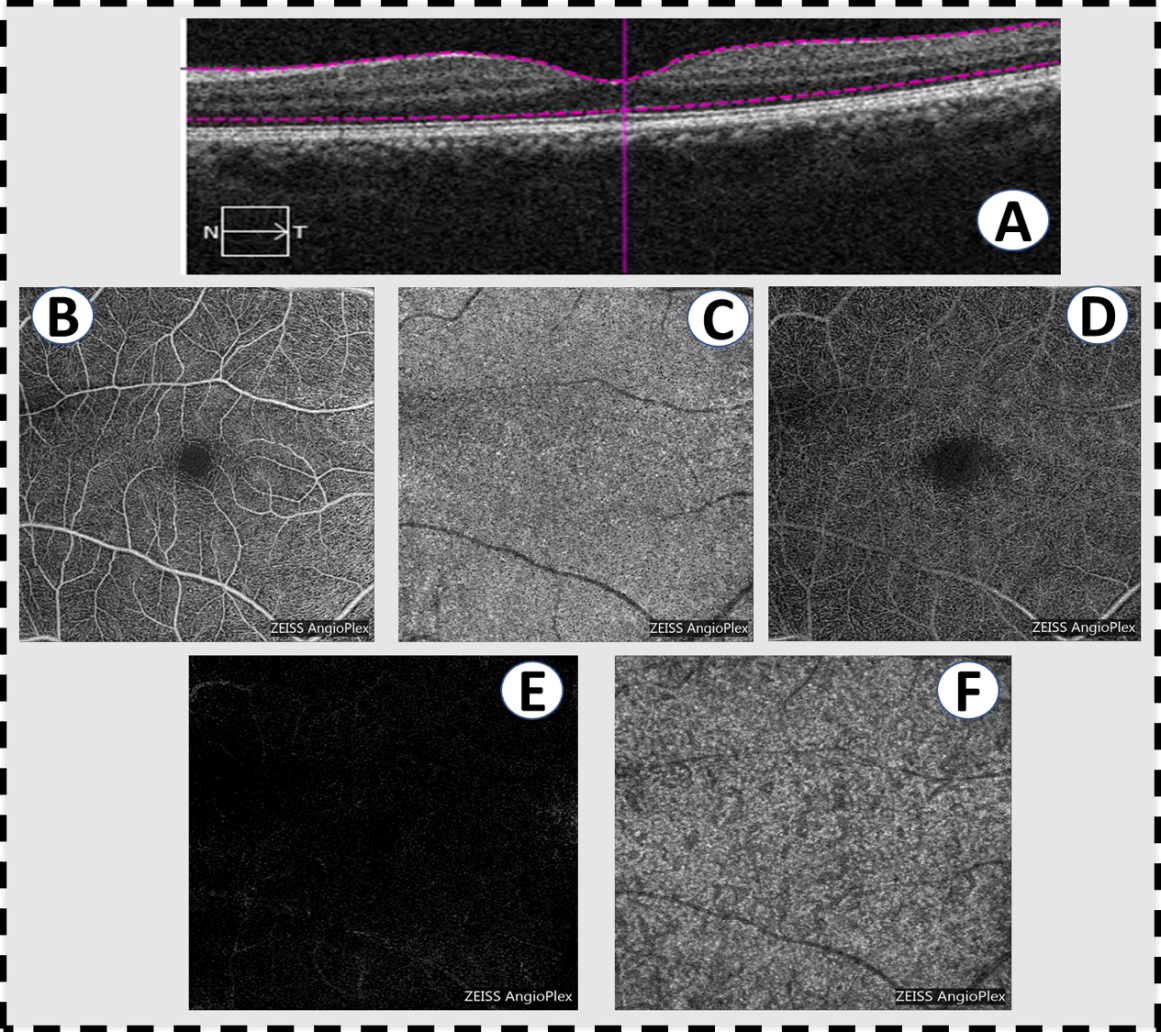
Optical coherence tomography angiography scans in a normal patient. (**A**) A horizontal slice of a conventional OCT scan showing normal macular anatomy. (**B**) The superficial vascular plexus of the inner retina. (**C**) The choriocapillaris. (**D**) The deep vascular plexus of the middle retina. (**E**) The outer retina is avascular, hence the absence of retinal vessels in a normal eye. (**F**) The deeper choroid.

**Figure 6:**
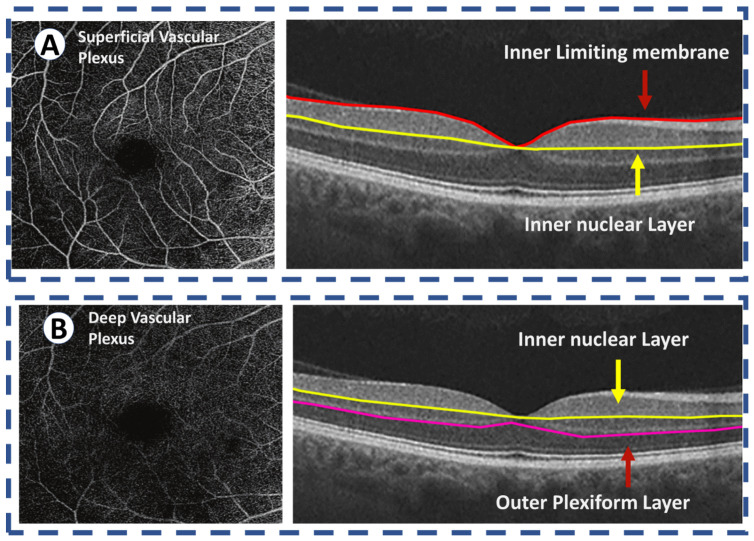
OCT angiography of the normal retina images the two vascular plexuses. (**A**) The superficial vascular plexus supplies the inner retina, defined as between the internal limiting membrane and the inner border of the inner nuclear layer. (**B**) The deep vascular plexus supplies the middle retina, defined as between the inner border nuclear layer and the outer border of the outer plexiform layer. The outer retina is avascular and receives its blood supply from the choriocapillaris.

In addition to evaluating the DR using OCT, OCTA is another relatively fast and noninvasive way of doing this. Figure 4 shows different OCT angiograms across varying severities of DR against healthy retina. In addition, the foveal microvasculature can be examined using this imaging modality (Figure 5) [35,36,37,38,39,40,41]. An OCT imaging sequence is processed with OCTA’s motion contrast method. These images are proceeded for obtaining perfusion maps without requiring extrinsic dye injection. OCTA enables measurement of vascular metrics. These metrics are shown to be closely consistent with histology [42,43]. In addition, it is correlated with other in vivo imaging modalities [38,39,44,45]. Ophthalmologists use deep vascular plexus (DVP) and superficial vascular plexus (SVP) of OCTA, which are defined as between the the inner border of the inner nuclear layer and internal limiting membrane, and between the inner border nuclear layer and the outer border of the outer plexiform layer, respectively, as shown in Figure 6; to detect and evaluate DR. One of the important observed findings in the DR is expansion of the foveal avascular zone (FAZ). Multiple studies have studied methods of measuring the foveal avascular zone (FAZ). The loss of capillaries from this region has been linked to substantial visual damage [46,47]. In addition, FAZ enlargement is noticed in sickle cell retinopathy and branch retinal vein occlusion [47,48,49,50,51,52]. Therefore, evaluation and visual assessment of FAZ is essential to determining macular perfusion and the degree of retinal damage. OCTA and IVFA measurements for FAZ dimensions have been found to be different [49,50,51]. Many recent studies have evaluated the role of OCTA in evaluation of FAZ dimensions [36,37,38,39,41]. To measure FAZ dimensions, one additional step is required because axial length measurement is required to account for different retinal enlargement [53,54]. This step is important to ensure the accurate calculations and is not always available. With OCTA, several publications have measured metrics in healthy retina and DR without axial length correction. These studies calculated the acircularity index, a metric in conjunction with adaptive optics imaging for quantifying irregularities in the FAZ, as well as the axis ratio of the FAZ [55]. Methods such as these can be used as biomarkers to identify vascular changes before the development of funduscopically visible DR and may help determine the severity level of DR.

### 3.4. Color Fundus Photography

Color FP uses seven standard fields (SSFs) in grading of the DR disease severity [20]. The use of SSFs is reliable and accurate; however, it is considered impractical as it requires high labor, more specialized photographers, and photograph interpreters. In addition, highly complicated photography equipment is needed. Although ophthalmoscopy is the most commonly utilized approach for monitoring DR, it requires specialized eyecare providers to produce highly sensitive results. Therefore, using SSFs color photography is generally considered more sensitive [56,57]. To analyze large volumes of available data, multiple systems have been developed. These systems still may be impractical for screening for DR as they are sophisticated and require highly skilled eye care providers and imaging technicians. The first system is the Joslin vision network [58]. It was found that there is a great agreement between three-field and digital-video color fundus photographs in the determination of DR severity and the rate of referral to highly specialized ophthalmologists for more clinical evaluation. In addition, results of Joslin vision network imaging have been greatly matched by the eye examination by retina specialists [59]. The Inoveon DR system recorded SSF color photography images on both 35 mm film and on proprietary systems [60]. The results were highly sensitive and specific. Although this system is highly accurate in DR referral decisions, it requires pupillary dilation and is expensive, hence it is not commonly used as a screening procedure. The DigiScope is a semiautomated system that evaluates visual acuity, acquires fundus images, and transmits the data through telephone lines to a remote reading center [61]. The results of recent studies are promising; however, more studies are needed to evaluate the test’s usefulness and accuracy. Diabetic patients were also evaluated by single-field FP. An ophthalmologist evaluated images of single-field digital monochromatic nonmydriatic photography (SNMDP) of both a non-pharmacologically dilated pupil and a pharmacologically dilated one using ophthalmoscopy, and then $30^{\circ }$ color stereoscopic photographs were obtained in SSFs [62]. The results of both SNMDP and SSFs showed excellent agreement regarding the degree of DR and the rate of referral. In addition, the SNMDP compared with SSFs had a sensitivity of 78% and a specificity of 86%, respectively. In comparison with SSFs, SNMDP outperformed ophthalmoscopy through pharmacologically dilated pupils. The sensitivity and specificity for SNMDP are 100% and 71%, respectively, when compared with direct ophthalmoscopy. SNMDP diagnosed all patients identified by ophthalmoscopy for referral. In multiple studies, SNMDP has been shown to be superior to dilated ophthalmoscopy [63,64]. We need to highlight that single-field photography cannot substitute the comprehensive ophthalmic examination. However, there are many well-designed comparative studies that proposed that single-field FP can be used initially for evaluation of DR by identifying patients with retinopathy before referral to ophthalmic assessment and management.

It is effective and practical because it is easy to use, convenient, affordable, and capable of detecting retinopathy, as one image is only required. Most importantly, it is impractical to manually evaluate these biomarkers, even in a dedicated ophthalmic reading center, because the sheer volume of imaging data exceeds capacities of human readers. Therefore, the future of ophthalmology involves automation of image data evaluation as seen by the large number of studies in this field on the automated segmentation in OCT [65,66,67,68] or automated detection of signs of DR in color FP [66,69,70,71]. Other computational methods using deep learning (DL) and artificial intelligence (AI) have been proposed by many of the recent studies [65,70,72,73,74], which is the new future of the medicine dealing with these diseases [75].

## 4. Literature on CAD Systems for DR Diagnosis and Grading

**Figure 7:**
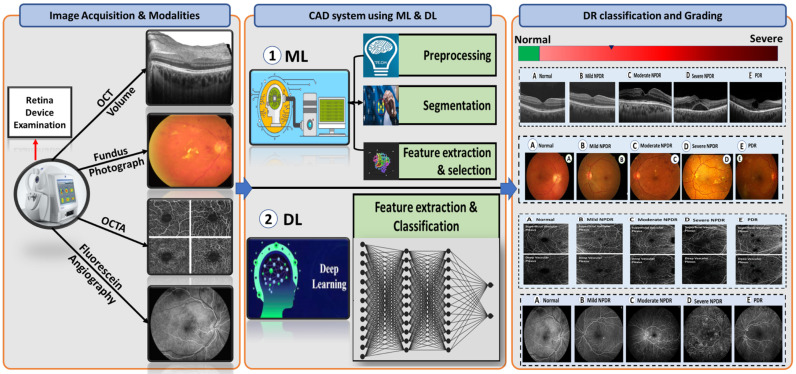
A flow diagram for a generic computer-assisted diagnostic (CAD) for DR classification. Typically, it starts with the image acquisition for possible different retinal modalities (FP, OCT, and OCTA) (**left panel**). Then, it applyies prepossessing and segmentation techniques on these modalities as well as applies ML methods and DL approaches (**middle panel**). Finally, the system makes a decision and diagnoses or grades DR based on the extracted features from different retinal modalities (**right panel**).

We provide in-depth analysis and present research and developments on identifying and diagnosing DR using a comparative analysis of different imaging modalities. There have been numerous tools and databases developed for the treatment of DR disease. A review of the various image modalities used in CAD research and applications is part of evaluating the work that uses images as data. Our objective is to present data from research concerning DR disease using the different image modalities. Figure 7 introduces an overview of the flow of a generic CAD system for DR classification. Typically, it starts with image acquisition for different image modalities from retina device examination. Then, the CAD system either works on applying different segmentation approaches to segment different lesions related to DR disease and extracting the features using ML-based methods or works on applying DCNN convolutional layers and softmax to extract features and make the DR classification. Finally, by applying DL/ML approaches on these images, the system can decide and grade DR into one of the following grades: healthy retina, NPDR, mild NPDR, moderate NPDR, severe NPDR, and PDR.

An examination of two publicly available databases, PubMed and IEEE Xplore, was conducted for this review. Choosing the databases was based on their accessibility, quality, and availability. Based on our literature review, we considered all relevant journal articles and conference papers up to February 2022. The published articles in the last eight years (from 2015 to 2022) on this topic reveal that AI-assisted DR diagnosis has progressed significantly.

The field of ocular imaging has made significant advances over the past century and it has emerged as a vital aspect of managing ocular disease and clinical management of patients in ophthalmology. A substantial amount of research and development has been conducted on CAD since the early 1980s, based on radiology images and medical images. In this review, we summarize the literature for early detection techniques for DR based on a combination of image processing, ML, and DL approaches, as seen in Table 2.

### 4.1. CAD System Based on Machine Learning Techniques

In this review, there are some ML algorithms used to diagnose and grade DR. Some of these ML algorithms rely on extracting hand-crafted features from image modality (e.g., texture and shape features [138,139,140,141,142,143,144,145]), as well as employing feature reduction techniques (i.e., linear discriminant analysis (LDA)-based feature selection, minimum redundancy maximum relevance (MRMR) feature selection [146], and principal component analysis (PCA) [147]). In addition, some of the other ML algorithms used in the classification and the prediction of the disease (i.e., k-nearest neighbor (KNN), support vector machine (SVM), logistic regression (LR), gradient boosting tree (XGBoost), artificial neural network (ANN), random forest (RF), and decision trees (DT)). These ML methods can use different imaging techniques for eye examination (i.e., FA, FP, OCT, and OCTA) to detect and grade DR. For example, Liu et al. [126] developed a CAD system that differentiates between healthy eye and DR using OCTA images. They applied the discrete wavelet transform on SVP, DVP, and retinal vascular network (RVN) images to extract the texture features. Then, they fit four classification models, namely, LR, LR with the elastic net penalty (LR_En), SVM, and XGBoost. They achieved 82% accuracy and AUC of 83%. Other ML-based CAD systems use FP images to detect or grade DR [76,78,80,81,82,83,84,88]. Most of them utilized the different ML classifier models fed with extracted features from FP images.

Several other ML-based methods have been designed to use the OCT and OCTA images to diagnose and grade DR, as proposed in [92,127,133,148,149,150]. Eltanboly et al. [92] designed a CAD method for DR diagnosis using OCT images. In this study, the CAD system started with segmenting twelve retinal layers from OCT images using an adaptive shape model based on Markov–Gibbs random field (MGRF) [151]. Then, they started working on extracting morphological and texture features from segmented layers. Finally, they applied an autoencoder classifier to classify the OCT images into normal and DR. Another study [133] introduced a CAD system to differentiate between DR cases and healthy retina based on 3D-OCT images. This system starts by segmenting the OCT into twelve layers and then extracts novel texture features (i.e., MGRF) from these segmented layers. Lastly, an NN is applied at the end of the system with a majority-voting schema to obtain the final diagnosis of DR. Sandhu et al. [118] introduced a CAD system that utilized OCT and OCTA images in addition to clinical information to diagnose DR. This system worked on extracting morphological and texture features from OCT and OCTA images and integrating them with the clinical information. Then, these features were fed into a random forest classifier to obtain the final diagnosis of DR. An automated method to analyze OCT volumes was presented by researchers [152] for DR diagnosis. First, they filter the images properly using specialized masks, and after registering, they create anatomically identical ROIs from different OCT images. In a subsequent step, stochastic gradient descent optimization is used to compute efficient B-spline transformations. Based on 105 subjects’ experiments, they were able to detect DR.

### 4.2. CAD System Based on Deep Learning Techniques

Most of the literature nowadays uses the current-state of-the-art DL techniques for diagnosing and grading DR using the different image modalities. Furthermore, DL demonstrated promising results in segmenting DR lesions in an automated fashion. In addition, DL can be applied on OCT images to extract the retinal layers and some related lesions to DR disease. For example, recently, Jancy et al. [153] worked on detecting the hard exudates from different retinal image modalities using DCNN. Holmberg et al. [154] used a pretrained DCNN (i.e., U-Net) to extract the retinal OCT layers to calculate the thickness of layers. Another system [155] used a DL technique, which was composed of a multi-scaled encoder–decoder neural network for segmenting the avascular zone area presented on the OCTA images for DR diagnosis. Quellec et al. [94] introduced a DL approach depending on ConvNets and the backpropagation method on two datasets from FP to detect four lesions in DR, namely, exudates, microaneurysms, hemorrhages, and cotton-wool spots. Sayres et al. [110] trained the Inception V4 model on a large dataset from FP to grade DR into five grades (healthy eye, NPDR, moderate NPDR, severe NPDR, and PDR). Hua et al. [156] used a DL approach to identify DR progression risk from FP images. For that, they designed a DCNN called Tri-SDN with a pretrained ResNet50 and applied it on baseline and follow-up information of FP images. Then, they applied ten-fold cross-validation to calculate the performance model on the extracted features from FP images and numerical risk factors. Abramoff et al. [65] used a public dataset sourced from FP images to diagnose DR. Their method applied the DL approach to identify DR, which achieved high efficiency and can be used as a predicting tool to reduce the risk of vision loss. Multiple other studies [93,94,95,96,98,100,103,104,106,109] used pretrained CNN approaches to diagnose and grade DR using FP images. Their approaches are called GoggleNet, AlexNet, ResNet50, ResNet 101, ConvNets, and VGG19. In addition, other studies applied DL approaches on OCT and OCTA images [113,114,115,116,121,131]. All pretrained CNN networks are dealt with as a blackbox, where the image of a given modality (i.e., FP, OCT, and OCTA) represents the input to the network architecture. Then, DL networks use the convolutional layers to extract different sizes of the feature maps. Then, a fully-connected layer with a softmax layer will work as a classifier to detect/diagnose or grade DR. Table 2 describes previous studies from 2015 to 2022 that used DL and ML methods for DR classification/grading.

## 5. Discussion and Future Directions

In this review, we have described, qualitatively and quantitatively, how ML/DL can be used for DR diagnosis and grading. It may be feasible to use a computer-aided automated DR assessment method in place of manual assessment, especially for rural and semi-urban populations without ready access to qualified ophthalmologists. To the best of our knowledge, this is the first review article that focuses on diagnosing and grading DR using different imaging modalities (i.e., FA, FP, OCT, and OCTA). We selected our reviewed publication articles based on the most visited databases (i.e., PubMed and IEEE Xplore). Our study revealed that DL methods are increasingly popular today, as opposed to ML approaches. In addition, we found that FP images are more popular in diagnosing and grading DR than other imaging modalities, such as OCT and OCTA. This review article is intended to conclude the review of a variety of disciplines such as ML, DL, computer vision, medical image analysis, and different imaging modalities that converge in this field. The following subsection will discuss the future research areas, challenges, and research gaps. We also offer a suggested solution for the researchers to start resolving these gaps.

### 5.1. Future Research Areas and Challenges

Researchers have extensive possibilities to develop intelligent, automated early detection and grading systems for DR that could provide medical professionals with clinical decision support systems. Based on our in-depth review of existing works, we found some shortcomings that could be improved to enhance existing CAD-based systems for early DR diagnosis and grading. First, the retinal images may differ from one another in terms of image dimensions, contrast, illumination, light incidence angle, etc., due to various camera settings. Training and testing are typically performed on a single dataset. In order to work with data from a variety of imaging machines and patient demographics, it is necessary to build robust models and verify cross-data. Among the possibilities is the use of neural-style transfer models [157]. Second, in order to use CNN models with supervised DL and more complex architectures, it is necessary to have thousands of correctly labeled FP, OCT, OCTA, and FA images with annotations at both pixel and image levels. Obtaining such images is an expensive process, requiring the assistance of specialists. To solve this issue, researchers can use semi-supervised learning such as generative adversarial networks (GANs), which can learn from limited data. Third, currently, the majority of FP images used in existing publications are captured with expensive fundoscopy devices. Thus, the need to utilize portable, low-cost technologies such as smartphones that can capture FP images is highly recommended. Mobile-based early DR screening systems may have a strong potential, especially for the elderly. This could potentially lower costs and promote remote screening without requiring direct contact with patients. Fourth, the researchers can use different modalities of the eye, which will be helpful in the diagnosis and grading of DR. Therefore, the concatenation of the features extracted from each modality from the eye can be fed into any traditional ML or DL algorithm and produce promising results. Fifth, there are currently more studies in the literature that use only the imaging data for early diagnosis and grading DR. There has been little work carried out on the association between patient clinical data and imaging findings (e.g., age, blood sugar level, gender, and blood pressure). Hence, there is a need to explore different imaging modalities and correlate their findings with their clinical demographics in order to improve the accuracy of diagnosing and grading DR, as well as potentially to enhance other retinal conditions. Sixth, retina specialists have demonstrated heterogeneity in their diagnostic decision-making, which is a major problem. As a result, ophthalmic modalities images are interpreted by experts with varying degrees of precision, which may lead to bias during the model training. Lastly, most publicly available datasets have few annotated images because manual annotation is time-consuming. Therefore, the goal is to develop better methods to collect clinical annotations that can be applied to different image modalities, such as the localization of exudates and retinal hemorrhages.

### 5.2. Research Gap

Survey papers on diagnosing and grading DR are frequently limited to discussion of the ability of different modalities to distinguish between normal and different categories of DR [14,15,158]. These survey articles consider the use of ML/DL on one specific modality (e.g., FP) to grade DR, and failed to analyze other different modalities in recognizing DR and its grades. Thus, our survey article, to the best of our knowledge, is the first one to address and discuss the use of ML and DL approaches on different imaging modalities and their clinical and demographic data for DR grading. We investigate the promising results for DR grading when applying the ML and DL approaches on OCT, OCTA, FP, and clinical information of the patients. As a result, a number of clinical studies have demonstrated the benefits and effectiveness of the application of DL and ML methodologies to retinal imaging assessment. Nevertheless, there are numerous shortcomings with DL approaches in addition to CAD systems, outlined as follows, along with possible improvements. First, for evaluating the performance of DL models, there are no standardized statistical measures. Most recent works have used only the different evaluation matrices such as accuracy, sensitivity, specificity, AUC, F1-score, and kappa score. Therefore, until now, the performance of DL for diagnosing diseases has been challenging to compare. Second, in the near future, it might be possible, and will be helpful for researchers, to include more public datasets that contain different imaging modalities, such as FP, OCT, and OCTA, in addition to clinical and demographic information to more precisely detect and diagnose DR in a more precise manner. Third, other related retina diseases may have different grades of DR characteristics based on the lesions detected in different image modalities. The goal then must be to build fully automated CAD systems based on distinguishing between DR disease and other non-DR diseases based on DL or ML approaches. Fourth, from the previous point, the segmentation-based DL approaches for detecting different lesions are a vital and essential step in the computer vision field. Therefore, testing should be conducted for the detection of many lesions present in FP, OCT, and OCTA images. Fifth, although this review article looks at a broad range of DR diseases, CAD systems are not considered for many other conditions that could be investigated in future studies. Lastly, several DL approaches are concerned with the computational complexity of identifying benchmark datasets when dealing with growing numbers of patients during the following years.

## 6. Conclusions

The purpose of the survey was to summarize the current developments in ML/DL algorithm models for diagnosing and grading diabetic retinal diseases by different imaging modalities. In this review article, we introduced, at the beginning, an overview of DR disease and its grading, including mild NPDR, moderate NPDR, severe NPDR, and PDR. Then, we discussed the different image modalities (i.e., FP, OCT, and OCTA) used in the diagnoses and grading of DR. In addition, a systematic review of the most recent publications on CAD-based methods for the detection and grading of DR was conducted, including traditional image processing as well as ML- to DL-based methods. We provided an overview of the methodology, number of DR grades identified, system performance, and database information for each work introduced in this survey. This paper’s most important contribution is the discussion of the advantages and challenges of existing methodologies for developing an automated and robust methodology for detecting and grading DR. After that, we discussed the future direction and research plans for how the most recent, state-of-the-art DL architectures work on detecting DR early. We identified the major obstacles associated with the development of DL-based approaches and introduced solutions for diagnosing DR based on integrating the four modalities ML features in addition to the clinical and demographic information for each patient. Finally, we also offered a future direction of using smartphone-based diagnosis of DR, which potentially lowers costs and promotes remote screening without the need for direct contact with the patients.

## Figures and Tables

**Table 2:** Recent studies for early detection and grading of DR based on a combination of image processing, ML, and DL approaches.

Study	Methodology	# of Grades	System Performance	Dataset Info.
Welikala et al. [76], 2015	Implemented a method that segments new vessels from FP images, then applied SVM on selected morphological features obtained from a genetic algorithm	Differentiated between normal and PDR	Sensitivity was 91.83% and specificity was 96%, while AUC was 96.93%	60 FP images from MESSIDOR [77] and St, Thomas’ Hospital ophthalmology department
Prasad et al. [78], 2015	Developed a method that used a back propagation neural network and PCA with extracted features from some morphological operations	Differentiated between normal and DR	Sensitivity and specificity were 97.8% and 97.5%, respectively; accuracy was 97.75%	Publicly available 89 FP images from DIARETDB1 [79]
Mahendran et al. [80], 2015	Introduced an SVM with probabilistic neural network and neighborhood-based segmentation technique to automatically detect FP lesions exudates	Differentiated between normal, moderate NPDR, and severe NPDR	Overall accuracy of SVM and neural network were 97.8% and 94.7%, respectively.	Publicly available 1200 FP images from MESSIDOR dataset
Bhatkar et al. [81], 2015	Introduced a multi-layer perception neural network with features extracted from discrete cosine transform	Differentiated between normal and DR	Overall accuracy was 100%	130 FP images from DIARETDB0 dataset
Labhade et al. [82], 2016	Applied different ML models (SVM, RF, gradient boost, and AdaBoost) on extracted CLCM features from FP images	Differentiated between normal, mild NPDR, severe NPDR, and PDR	Accuracy of SVM was 88.71%, RF was 83.34%, gradient boost was 83.34%, and AdaBoost was 54.3%	1200 FP images from public Messidor dataset
Rahim et al. [83], 2016	Introduced an ML algorithm (SVM with RBF kernel) and a combination of fuzzy fuzzy image processing techniques and circular Hough transform	Differentiated between no DR, mild NPDR, moderate NPDR, severe NPDR, and PDR	SVM with RBF kernel: accuracy was 93%, specificity was 93.62%, and sensitivity was 92.45%	600 FP images from 300 patients collected at the Hospital Melaka, Malaysia
Bhatia et al. [84], 2016	Applied different ML algorithms on extracted lesions from FP (microaneurysms and exudates) and calculation of the optic disk diameter	Differentiated between normal and different severity levels of DR	Overall accuracy was 94% and F1-score was 93%	1200 FP images from public MESSIDOR dataset
Gulshan et al. [85], 2016	Designed a DCNN for automated detection and diagnosis of DR and DME using three different datasets from FP images	Differentiated between normal, different levels of DR and DME	The AUC was 99.1% for EyePACS-1 and The AUC was 99% for Messidor-2	128,175 FP + 9963 FP from EyePACS-1 + 1748 from Messidor-2
Colas et al. [86], 2016	Built algorithm to detect the anomalies locations for FP images	Grading based on ICDR severity scale	The AUC was 94.6%, sensitivity was 96.2%, and 66.6% specificity	70,000 FP images for training + 15,000 FP images for testing
Ghosh et al. [87], 2017	Designed a DCNN model to identify different lessions in FP images such as micro-aneurysms and hemorrhages	Grading based on ICDR severity scale	95% accuracy for binary classification and 85% accuracy for 5-class classification	88,702 FP images from EyePACS dataset
Islam et al. [88], 2017	Designed an ML algorithm that used the bag of words model to identify some lesions in FP images	Differentiated between normal and DR	94.4% accuracy, 94% precision, 94% F1-score, and 95% AUC	180 FP images from four public dataset
Carrera et al. [89], 2017	Implemented CAD system based on SVM model and extracted features from blood vessels, microaneurysms, and hard exudates	Differentiated between four grades from NPDR	Accuracy of SVM was 92.4%, specificity was 97.4%, AUC was 93.8%	400 FP images from public Messidor dataset
Somasundaram et al. [90], 2017	Designed an ML bagging ensemble classifier and t-distributed stochastic neighbor embedding	Differentiated between NPDR and PDR.	ML-BEC approach accomplishes accuracies of 40% and 49% for DR detection	89 FP images from public dataset [91]
Eltanboly et al. [92], 2017	Implemented deep fusion classification network (DFCN) with extracted morphological features from segmented retina layers	Differentiated between normal and DR	Accuracy was 92%, specificity was 100%, sensitivity was 83%	52 OCT images from University of Louisville, USA
Takahashi et al. [93], 2017	Modified GoggleNet DCNN approach	Differentiated between NPDR, severe NPDR, and PDR	The grading accuracy was 81%	9939 FP images from Jichi Medical University
Quellec et al. [94], 2017	A DL approach depending on ConvNets and the backpropagation method	Grading based on ICDR severity scale	Detection performance was 95.4% and 94.9% on two different datasets	90,000 FP images from Public and private dataset
Ting et al. [95], 2017	Designed a DCNN pretrained to diagnose and grade DR using FP images	Differentiated between PDR, vision-threatening DR, glaucoma, and AMD	AUC for PDR was 0.93 and AUC for vision-threatening DR was 0.95	494,661 FP images from Singapore National DR Program
Wang et al. [96], 2017	Designed a CNN called Zoom-in-Net to identify suspicious areas using the created attention maps	Grading based on ICDR severity scale	AUC for Messidor dataset was 0.95 and AUC for EyePACS dataset was 0.92	1200 FP images from Messidor + 89,000 FP images from EyePACS public dataset
Eladawi et al. [97], 2018	Designed system used MGRF to segment blood vessels from SVP and DVP, then, used SVM with local features extracted	Differentiated between healthy eye and DR	Accuracy was 97.3%, specificity of 96.4%, sensitivity was 97.9%, and AUC was 97.2%	105 OCTA images from the University of Louisville, USA
Dutta et al. [98], 2018	Designed backpropagation NN, DNN, and CNN (VGGNet)	Differentiated between mild NPDR, moderate NPDR, severe NPDR, and PDR	86.3% accuracy for DNN, 78.3% accuracy for VGGNet, 42% accuracy for backpropagation NN	2000 FP images selected from public dataset
Eltanboly et al. [99], 2018	Introduced a stacked non-negativity constraint autoencoder and fed it with extracted features from the segmented retinal OCT layers	Differentiated between healthy, early DR, mild, or moderate DR	Using LOSO, accuracy of the first stage was 93%, and the second stage was 98%	74 OCT images from the University of Louisville, USA
Zhang et al. [100], 2018	Designed DCNN model called DR-Net with a new adaptive cross-entropy loss	Grading based on ICDR severity scale	The overall accuracy was 82.10%, and kappa score was 66%	88,702 FP images from EyePACS dataset
Costa et al. [101], 2018	Developed an ML technique depending on new multiple instances learning for DR detection using FP images	Grading DR based on ICDR severity scale	Messidor: AUC was 90%, DR1: AUC was 93%, DR2: AUC was 96%	1200 FP from Messidor dataset + 1077 FP from DR1 and DR2 dataset [102]
Chakrabarty et al. [103], 2018	Designed a DL approach and applied it on enhanced high-resolution FP images	Differentiated between healthy eye and DR	Accuracy of 91.67%, sensitivity of 100%, and precision of 100%	30 high-resolution FP images
Kwasigroch et al. [104], 2018	Proposed a CAD system based on a DCNN approach called VGG-D	Grading based on ICDR severity scale	Accuracy was 81.7%, specificity was 50.5%, and sensitivity was 89.5%	Over 88,000 FP images from EyePACKS [105]
Li et al. [106], 2019	Proposed a CAD system based on a deep transfer learning approach called Inception-v3	Grading based on ICDR severity scale	Accuracy of 93.49%, sensitivity of 96.93%, specificity of 93.45%, and AUC of 0.99	19,233 FP images from public Messidor-2 dataset
Nagasawa et al. [107], 2019	Proposed a CAD system based on a deep transfer learning approach called Inception-v3	Differentiated between non-PDR and PDR	AUC of 96.9%, sensitivity of 94.7%, and specificity of 97.2%	378 FP images from Tokushima University and Saneikai Tsukazaki Hospitals
Metan et al. [108], 2019	Proposed a CAD system based on ResNet with shallow and deep skip connections	Grading based on ICDR severity scale	The performance accuracy of system was 81%	88,702 FP images from EyePACKS [105]
Qummar et al. [109], 2019	Designed five different DCNNs (Resnet50, Inceptionv3, Xception, Dense121, Dense169)	Grading based on ICDR severity scale	Accuracy of 80.80%, recall of 51.50%, specificity of 86.72%, and F1-score of 53.74%	88,702 FP images from public EyePACKS [105]
Sayres et al. [110], 2019	Trained the Inception V4 model on a large dataset from FP	Grading based on ICDR severity scale	The overall accuracy was 88.40%	88,702 FP images from public EyePACKS [105]
Sengupta et al. [111], 2019	Trained a DCNN called InceptionV3 model on a large dataset from FP	Grading based on ICDR severity scale	The overall accuracy was 90.40%, specificity of 91.94%, and sensitivity of 90%	88,702 FP images from public EyePACKS [105], and MESSIDOR1 [77]
Hathwar et al. [112], 2019	Designed pretrained CNN called Xception-TL to diagnose and grade DR using FP images	Grading DR based on ICDR severity scale	quadratic weighted kappa score was 88% for grading DR; sensitivity of 94.3% for DR vs. No DR	35,124 FP images from EyePACS and 413 FP from IDRiD dataset
Li et al. [113], 2019	Developed and designed a DCNN model called OCTD_Net for early detection of DR	Differentiated between healthy eye, and grade 0 DR, and Grade 1 DR	Accuracy was 92%, specificity was 95%, and sensitivity was 92%	4168 OCT images from Wenzhou Medical University
Heisler et al. [114], 2020	Designed DCNN models based on VGG19, ResNet50, and DenseNet and ensembled using majority soft voting and stacking techniques	Grading based on ICDR severity scale	The overall accuracy for VGG19 was 92% and 90% for the majority soft voting and stacking methods, respectively	463 volumes from OCT and OCTA images from 380 eyes
Alam et al. [115], 2020	Introduced an SVM model, which is fed with six different features extracted from OCTA images	Differentiated between normal and three stages from NPDR	Accuracy of 94.41% for control vs. DR; Accuracy of 92.96% for control vs. NPDR specificity	120 OCTA images from 60 patients
Zang et al. [116], 2020	Introduced a DCNN called DcardNet with adaptive label smoothing to suppress overfitting using en-face OCT and OCTA images	Differentiated between healthy, mild NPDR, moderate NPDR, severe NPDR, and PDR	Accuracies of 95.7%, 85.0%, and 71% for three-level classifiction	303 OCT and OCTA images from 250 participants
Ghazal et al. [117], 2020	Introduced a CAD system based on a novel seven-CNN model with SVM to early diagnose DR	Differentiated between healthy and DR	Accuracies of 94%, recall of 100%, and specificity of 88%	52 OCT images from University of Louisville, USA
Sandhu et al. [118], 2020	Introduced a CAD system based on a random forest classifier and fed with extracted features from OCT and OCTA images in addition to clinical markers	Differentiated between healthy, mild NPDR, and moderate NPDR	Accuracy of 96%, sensitivity of 100%, specificity of 94.1%, and AUC of 0.96	111 volumes from OCT and OCTA images, University of Louisville, USA
Narayanan et al. [119], 2020	Established a hybrid ML algorithm with CNN and PCA to detect and grade DR	Grading DR based on ICDR severity scale	AUC was 98.5%, and the overall accuracy was 98.4%	3662 FP images from APTOS 2019
Shankar et al. [120], 2020	Introduced DL model to diagnose and grade DR by applying histogram-based segmentation to segment the ROI regions in FP images and then applying synergic DL model	Grading DR based on ICDR severity scale	Overall accuracy was 99.28%, sensitivity was 98%, and specificity was 99%	3662 FP images from APTOS 2019
Ryu et al. [121], 2021	Developed fully automated system based on CNN model called ResNet101 for early detection of DR using OCTA images	Grading based on ICDR severity scale	The range of AUC was 93% to 97% for detecting DR, while accuracy was 90% to 95%, and sensitivity was 91% to 98%	OCTA images from 496 eyes
He et al. [122], 2021	Developed an attention module with global attention block (GAB) and with a backbone network to identify different lesions in different DR grades	Grading DR based on ICDR severity scale	Messidor: accuracy of 84.08% and 0.8723 kappa score	1200 FP from Messidor + 13,673 FP from DDR DataSet [123] + 88,702 FP from EyePACS
Saeed et al. [124], 2021	Developed a CAD system based on two pretrained DCNN for DR grading using FP images	Grading DR based on ICDR severity scale	EyePACS: accuracy of 99.73% and AUC of 89%	1200 FP from Messidor + 88,702 FP from EyePACS
Wang et al. [125], 2021	Developed a CAD system based on two pretrained DCNN for DR grading using FP images	Grading DR based on ICDR severity scale	AUC of 94.3%, kappa score of 69.6%, and F1-score of 85.54%	22,948 FP images from EyePACS and Peking Union Medical College Hospital
Liu et al. [126], 2021	Introduced four ML algorithms (LR, LR-EN, SVM, and XGBoost) fed with extracted features from a discrete wavelet transform	Differentiated between healthy and DR	LR-EN and LR had the highest accuracy of 82% and AUC of 83% and 84%, respectively.	246 OCTA images from 44 patients
Sharafeldeen et al. [127], 2021	Introduced a CAD system based on a fused NN and SVM model and fed with extracted texture and morphological features from OCT retinal layers	Differentiated between healthy and NPDR	Using LOSO, accuracy of 97.69%, sensitivity of 96.15%, specificity of 99.23%, and F1-score of 97.66%	260 OCT images from 130 patients
Hsieh et al. [128], 2021	Designed a two-DCNN Inception v4 and ResNet, the first for distinguishing between DR and RDR and the second for PDR	Differentiated between DR, RDR, and PDR	The AUCs for DR, RDR, and PDR were 0.955, 0.955 and 0.984, respectively	7524 FP and 31,612 FP images from EyePACS
Khan et al. [129], 2021	Designed a DCNN called VGG-NiN model that is a stacked layers from spatial pyramid pooling layer and VGG16 layers	Differentiated between DR, RDR, and PDR	The average AUC was 83.8, The average recall was 55.6, and the average F1-score was 59.6	88,702 FP images from EyePACS
Wang et al. [130], 2021	Analyzed OCTA images from SVP, DVP, and radial peripapillary capillary plexus images	Differentiated between DR, NPDR, and PDR	Sensitivity was 83.7%, and specificity was 78.3%	150 OCTA images from 105 diabetic patients
Abdelsalam et al. [131], 2021	Designed an ML method that used SVM with multifractal geometry and lacunarity parameters to diagnose early DR using OCTA images	Differentiated between normal and mild NPDR	Sensitivity was 100%, specificity was 97.3%, and precision was 96.8%	113 OCTA used for training and 67 OCTA for testing
Gao et al. [132], 2022	Designed three pretrained DCNN models called VGG16, ResNet50, and DenseNet for grading DR	Differentiated between DR, RDR, and PDR	The overall accuracies for VGG16, ResNet50, and DenseNet were 91.11%, 90.22%, and 90.87%, respectively	11,214 FA images from Xian and Ningbo dataset
Elsharkawy et al. [133], 2022	Introduced a CAD system based on an NN classifier and fed with extracted higher-order appearance features from OCT images	Differentiated between healthy and DR	Accuracies were 90.56%, 93.11%, and 96.88% using different k-folds cross validation	188 volumes from OCT images
Zia et al. [134], 2022	Introduced a hybrid system from DL (pretrained CNN, i.e., VGG VD-19 and Inception V3) and ML (cubic-SVM) to grade DR using FP images	Grading based on ICDR severity scale	Cubic-SVM: AUC of 99.80%, sensitivity of 96.4%, and precision of 96.4%	35,126 FP images from the Kaggle dataset
Zang et al. [116], 2022	Developed a DCNN to grade the different severity levels of DR using FP images with applying custom weight loss to solve unbalanced problems found in the dataset	Differentiated between healthy, mild NPDR, moderate NPDR, severe NPDR, and PDR	Accuracy of 92.49%, kappa score of 94.5%, while weighted average F1-score was 93%, recall 92%, precision 93%	5590 FP images from APTOS 2019! [135]
Tsai et al. [136], 2022	Designed three DCNNs to grade the different severity levels of DR using pretrained CNNs called ResNet101, DenseNet121, and Inception-v3	Differentiated between healthy, mild NPDR, moderate NPDR, severe NPDR, and PDR	Inception-v3 gave the highest accuracies of 84.64% and 83.80 for Kaggle test and Taiwanese dataset, respectively	88,702 FP images from EyePACS + local Taiwanese dataset of 4038 FP images
Das et al. [137], 2022	Built DCNN based on genetic algorithm based technique and used SVM for final classification	Differentiated between healthy, mild NPDR, severe NPDR, and PDR	Overall accuracy of 98.67% and AUC of 99.33%	1200 FP images from public Messidor [77]

## Data Availability

No data available.

## References

[1] Duh E.J., Sun J.K., Stitt A.W. (2017). Diabetic retinopathy: Current understanding, mechanisms, and treatment strategies. JCI Insight.

[2] Schwartz S.S., Epstein S., Corkey B.E., Grant S.F., Gavin J.R., Aguilar R.B., Herman M.E. (2017). A unified pathophysiological construct of diabetes and its complications. Trends Endocrinol. Metab..

[3] Ruta L., Magliano D., Lemesurier R., Taylor H., Zimmet P., Shaw J. (2013). Prevalence of diabetic retinopathy in Type 2 diabetes in developing and developed countries. Diabet. Med..

[4] Lachin J.M., Genuth S., Nathan D.M., Zinman B., Rutledge B.N. (2008). Effect of glycemic exposure on the risk of microvascular complications in the diabetes control and complications trial—Revisited. Diabetes.

[5] Klein R., Klein B.E., Moss S.E., Cruickshanks K.J. (1998). The Wisconsin Epidemiologic Study of Diabetic Retinopathy: XVII: The 14-year incidence and progression of diabetic retinopathy and associated risk factors in type 1 diabetes. Ophthalmology.

[6] Hietala K., Forsblom C., Summanen P., Groop P.H. (2008). Heritability of proliferative diabetic retinopathy. Diabetes.

[7] Frank R.N., Keirn R.J., Kennedy A., Frank K.W. (1983). Galactose-induced retinal capillary basement membrane thickening: Prevention by Sorbinil. Investig. Ophthalmol. Vis. Sci..

[8] Engerman R.L., Kern T.S. (1987). Progression of incipient diabetic retinopathy during good glycemic control. Diabetes.

[9] Giugliano D., Ceriello A., Paolisso G. (1996). Oxidative stress and diabetic vascular complications. Diabetes Care.

[10] Sinclair S.H., Schwartz S.S. (2019). Diabetic retinopathy—An underdiagnosed and undertreated inflammatory, neuro-vascular complication of diabetes. Front. Endocrinol..

[11] Gerendas B.S., Bogunovic H., Sadeghipour A., Schlegl T., Langs G., Waldstein S.M., Schmidt-Erfurth U. (2017). Computational image analysis for prognosis determination in DME. Vis. Res..

[12] Aiello L., Gardner T., King G., Blanken-ship G., Cavallerano J., Ferris F. (1998). Diabetes Care (technical review). Diabetes Care.

[13] Soomro T.A., Gao J., Khan T., Hani A.F.M., Khan M.A., Paul M. (2017). Computerised approaches for the detection of diabetic retinopathy using retinal fundus images: A survey. Pattern Anal. Appl..

[14] Sarki R., Ahmed K., Wang H., Zhang Y. (2020). Automatic detection of diabetic eye disease through deep learning using fundus images: A survey. IEEE Access.

[15] Asiri N., Hussain M., Al Adel F., Alzaidi N. (2019). Deep learning based computer-aided diagnosis systems for diabetic retinopathy: A survey. Artif. Intell. Med..

[16] ETDRS Research Group (1991). Early photocoagulation for diabetic retinopathy. ETDRS report number 9. Ophthalmology.

[17] Klein R., Klein B.E., Moss S.E., Davis M.D., DeMets D.L. (1989). The Wisconsin Epidemiologic Study of Diabetic Retinopathy: X. Four-year incidence and progression of diabetic retinopathy when age at diagnosis is 30 years or more. Arch. Ophthalmol..

[18] Diabetic Retinopathy Study Research Group (1981). Diabetic retinopathy study report number 6. Design, methods, and baseline results. Report number 7. A modification of the Airlie House classification of diabetic retinopathy. Prepared by the diabetic retinopathy. Investig. Ophthalmol. Vis. Sci..

[19] Early Treatment Diabetic Retinopathy Study Research Group (1991). Classification of diabetic retinopathy from fluorescein angiograms: ETDRS report number 11. Ophthalmology.

[20] Early Treatment Diabetic Retinopathy Study Research Group (1991). Grading diabetic retinopathy from stereoscopic color fundus photographs—An extension of the modified Airlie House classification: ETDRS report number 10. Ophthalmology.

[21] Wilkinson C., Ferris F.L., Klein R.E., Lee P.P., Agardh C.D., Davis M., Dills D., Kampik A., Pararajasegaram R., Verdaguer J.T. (2003). Proposed international clinical diabetic retinopathy and diabetic macular edema disease severity scales. Ophthalmology.

[22] Ffytche T., Shilling J., Chisholm I., Federman J. (1980). Indications for fluorescein angiography in disease of the ocular fundus: A review. J. R. Soc. Med..

[23] Novotny H.R., Alvis D.L. (1961). A method of photographing fluorescence in circulating blood in the human retina. Circulation.

[24] Rabb M.F., Burton T.C., Schatz H., Yannuzzi L.A. (1978). Fluorescein angiography of the fundus: A schematic approach to interpretation. Surv. Ophthalmol..

[25] Mendis K.R., Balaratnasingam C., Yu P., Barry C.J., McAllister I.L., Cringle S.J., Yu D.Y. (2010). Correlation of histologic and clinical images to determine the diagnostic value of fluorescein angiography for studying retinal capillary detail. Investig. Ophthalmol. Vis. Sci..

[26] Balbino M., Silva G., Correia G.C.T.P. (2012). Anafilaxia com convulsões após angiografia com fluoresceína em paciente ambulatorial. Einstein.

[27] Johnson R.N., McDonald H.R., Schatz H. (1998). Rash, fever, and chills after intravenous fluorescein angiography. Am. J. Ophthalmol..

[28] Yannuzzi L.A., Rohrer K.T., Tindel L.J., Sobel R.S., Costanza M.A., Shields W., Zang E. (1986). Fluorescein angiography complication survey. Ophthalmology.

[29] Early Treatment Diabetic Retinopathy Study Research Group (1991). Fundus photographic risk factors for progression of diabetic retinopathy: ETDRS report number 12. Ophthalmology.

[30] Verdaguer T. (2001). Screening para retinopatia diabetica en Latino America. Resultados. Rev. Soc. Brasil Retina Vitreo.

[31] Fukuda M. (1983). Clinical arrangement of classification of diabetic retinopathy. Tohoku J. Exp. Med..

[32] Gyawali R., Toomey M., Stapleton F., Zangerl B., Dillon L., Keay L., Liew G., Jalbert I. (2021). Quality of the Australian National Health and Medical Research Council’s clinical practice guidelines for the management of diabetic retinopathy. Clin. Exp. Optom..

[33] Huang D., Swanson E.A., Lin C.P., Schuman J.S., Stinson W.G., Chang W., Hee M.R., Flotte T., Gregory K., Puliafito C.A. (1991). Optical coherence tomography. Science.

[34] Rivellese M., George A., Sulkes D., Reichel E., Puliafito C. (2000). Optical coherence tomography after laser photocoagulation for clinically significant macular edema. Ophthalmic Surgery Lasers Imaging Retin..

[35] Agemy S.A., Scripsema N.K., Shah C.M., Chui T., Garcia P.M., Lee J.G., Gentile R.C., Hsiao Y.S., Zhou Q., Ko T. (2015). Retinal vascular perfusion density mapping using optical coherence tomography angiography in normals and Diabetic Retinopathy patients. Retina.

[36] Di G., Weihong Y., Xiao Z., Zhikun Y., Xuan Z., Yi Q., Fangtian D. (2016). A morphological study of the foveal avascular zone in patients with diabetes mellitus using optical coherence tomography angiography. Graefe’s Arch. Clin. Exp. Ophthalmol..

[37] Freiberg F.J., Pfau M., Wons J., Wirth M.A., Becker M.D., Michels S. (2016). Optical coherence tomography angiography of the foveal avascular zone in diabetic retinopathy. Graefe’s Arch. Clin. Exp. Ophthalmol..

[38] Hwang T.S., Jia Y., Gao S.S., Bailey S.T., Lauer A.r.K., Flaxel C.J., Wilson D.J., Huang D. (2015). Optical coherence tomography angiography features of diabetic RETINOPATHY. Retina.

[39] Kim D.Y., Fingler J., Zawadzki R.J., Park S.S., Morse L.S., Schwartz D.M., Fraser S.E., Werner J.S. (2012). Noninvasive imaging of the foveal avascular zone with high-speed, phase-variance optical coherence tomography. Investig. Ophthalmol. Vis. Sci..

[40] Mastropasqua R., Di Antonio L., Di Staso S., Agnifili L., Di Gregorio A., Ciancaglini M., Mastropasqua L. (2015). Optical coherence tomography angiography in retinal vascular diseases and choroidal neovascularization. J. Ophthalmol..

[41] Takase N., Nozaki M., Kato A., Ozeki H., Yoshida M., Ogura Y. (2015). Enlargement of foveal avascular zone in diabetic eyes evaluated by en face optical coherence tomography angiography. Retina.

[42] Mammo Z., Balaratnasingam C.r., Yu P., Xu J., Heisler M., Mackenzie P., Merkur A.r., Kirker A.r., Albiani D., Freund K.B. (2015). Quantitative noninvasive angiography of the fovea centralis using speckle variance optical coherence tomography. Investig. Ophthalmol. Vis. Sci..

[43] Tan P.E.Z., Balaratnasingam C.r., Xu J., Mammo Z., Han S.X., Mackenzie P., Kirker A.r.W., Albiani D., Merkur A.r.B., Sarunic M.V. (2015). Quantitative comparison of retinal capillary images derived by speckle variance optical coherence tomography with histology. Investig. Ophthalmol. Vis. Sci..

[44] Mo S., Krawitz B., Efstathiadis E., Geyman L., Weitz R., Chui T.Y., Carroll J., Dubra A., Rosen R.B. (2016). Imaging foveal microvasculature: Optical coherence tomography angiography versus adaptive optics scanning light ophthalmoscope fluorescein angiography. Investig. Ophthalmol. Vis. Sci..

[45] Spaide R.F., Klancnik J.M., Cooney M.J. (2015). Retinal vascular layers imaged by fluorescein angiography and optical coherence tomography angiography. JAMA Ophthalmol..

[46] Arend O., Wolf S., Harris A., Reim M. (1995). The relationship of macular microcirculation to visual acuity in diabetic patients. Arch. Ophthalmol..

[47] Parodi M.B., Visintin F., Della Rupe P., Ravalico G. (1995). Foveal avascular zone in macular branch retinal vein occlusion. Int. Ophthalmol..

[48] Arend O., Wolf S., Jung F.r., Bertram B., Pöstgens H., Toonen H., Reim M. (1991). Retinal microcirculation in patients with diabetes mellitus: Dynamic and morphological analysis of perifoveal capillary network. Br. J. Ophthalmol..

[49] Bresnick G.H., Condit R., Syrjala S., Palta M., Groo A., Korth K. (1984). Abnormalities of the foveal avascular zone in DIABETIC RETINOPATHY. Arch. Ophthalmol..

[50] Conrath J., Giorgi R., Raccah D., Ridings B. (2005). Foveal avascular zone in diabetic retinopathy: Quantitative vs. qualitative assessment. Eye.

[51] Mansour A., Schachat A.R., Bodiford G., Haymond R. (1993). Foveal avascular zone in diabetes mellitus. Retina.

[52] Sanders R.J., Brown G.C., Rosenstein R.B., Magargal L. (1991). Foveal avascular zone diameter and sickle cell disease. Arch. Ophthalmol..

[53] Bennett A.G., Rudnicka A.R., Edgar D.F. (1994). Improvements on Littmann’s method of determining the size of retinal features by fundus photography. Graefe’s Arch. Clin. Exp. Ophthalmol..

[54] Popovic Z., Knutsson P., Thaung J., Owner-Petersen M., Sjöstrand J. (2011). Noninvasive imaging of human foveal capillary network using dual-conjugate adaptive optics. Investig. Ophthalmol. Vis. Sci..

[55] Tam J., Dhamdhere K.P., Tiruveedhula P., Manzanera S., Barez S., Bearse M.A., Adams A.J., Roorda A. (2011). Disruption of the retinal parafoveal capillary network in type 2 diabetes before the onset of DIABETIC RETINOPATHY. Investig. Ophthalmol. Vis. Sci..

[56] Hutchinson A., McIntosh A., Peters J., O’keeffe C., Khunti K., Baker R., Booth A. (2000). Effectiveness of screening and monitoring tests for diabetic retinopathy–a systematic review. Diabet. Med..

[57] Sussman E.J., Tsiaras W.G., Soper K.A. (1982). Diagnosis of diabetic eye disease. JAMA.

[58] Bursell S.E., Cavallerano J.D., Cavallerano A.A., Clermont A.C., Birkmire-Peters D., Aiello L.P., Aiello L.M., Joslin Vision Network Research Team (2001). Stereo nonmydriatic digital-video color retinal imaging compared with Early Treatment Diabetic Retinopathy Study seven standard field 35-mm stereo color photos for determining level of diabetic retinopathy. Ophthalmology.

[59] Cavallerano A.A., Cavallerano J.D., Katalinic P., Tolson A.M., Aiello L.P., Aiello L.M. (2003). Use of Joslin Vision Network digital-video nonmydriatic retinal imaging to assess diabetic retinopathy in a clinical program. Retina.

[60] Fransen S.R., Leonard-Martin T.C., Feuer W.J., Hildebrand P.L., Inoveon Health Research Group (2002). Clinical evaluation of patients with diabetic retinopathy: Accuracy of the Inoveon diabetic retinopathy-3DT system. Ophthalmology.

[61] Zeimer R., Zou S., Meeder T., Quinn K., Vitale S. (2002). A fundus camera dedicated to the screening of diabetic retinopathy in the primary-care physician’s office. Investig. Ophthalmol. Vis. Sci..

[62] Taylor D., Fisher J., Jacob J., Tooke J. (1999). The use of digital cameras in a mobile retinal screening environment. Diabet. Med..

[63] Pugh J.A., Jacobson J.M., Van Heuven W., Watters J.A., Tuley M.R., Lairson D.R., Lorimor R.J., Kapadia A.S., Velez R. (1993). Screening for diabetic retinopathy: The wide-angle retinal camera. Diabetes Care.

[64] Joannou J., Kalk W., Ntsepo S., Berzin M., Joffe B., Raal F., Sachs E., van der Merwe M., Wing J., Mahomed I. (1996). Screening for Diabetic retinopathy in South Africa with 60 retinal colour photography. J. Intern. Med..

[65] Abràmoff M.D., Lou Y., Erginay A., Clarida W., Amelon R., Folk J.C., Niemeijer M. (2016). Improved automated detection of diabetic retinopathy on a publicly available dataset through integration of deep learning. Investig. Ophthalmol. Vis. Sci..

[66] Chen X., Niemeijer M., Zhang L., Lee K., Abramoff M.D., Sonka M. (2012). Three-Dimensional Segmentation of Fluid-Associated Abnormalities in Retinal OCT: Probability Constrained Graph-Search-Graph-Cut. IEEE Trans. Med. Imaging.

[67] Sophie R., Lu N., Campochiaro P.A. (2015). Predictors of functional and anatomic outcomes in patients with diabetic macular edema treated with ranibizumab. Ophthalmology.

[68] Yohannan J., Bittencourt M., Sepah Y.J., Hatef E., Sophie R., Moradi A.R., Liu H., Ibrahim M., Do D.V., Coulantuoni E. (2013). Association of retinal sensitivity to integrity of photoreceptor inner/outer segment junction in patients with diabetic macular edema. Ophthalmology.

[69] Gerendas B.S., Waldstein S.M., Simader C., Deak G., Hajnajeeb B., Zhang L., Bogunovic H., Abramoff M.D., Kundi M., Sonka M. (2014). Three-dimensional automated choroidal volume assessment on standard spectral-domain optical coherence tomography and correlation with the level of diabetic macular edema. Am. J. Ophthalmol..

[70] Schlegl T., Waldstein S.M., Vogl W.D., Schmidt-Erfurth U., Langs G. (2015). Predicting semantic descriptions from medical images with convolutional neural networks. Inf. Process. Med. Imaging.

[71] Schmidt-Erfurth U., Waldstein S.M., Deak G.G., Kundi M., Simader C. (2015). Pigment epithelial detachment followed by retinal cystoid degeneration leads to vision loss in treatment of neovascular age-related macular degeneration. Ophthalmology.

[72] Ritter M., Simader C., Bolz M., Deák G.G., Mayr-Sponer U., Sayegh R., Kundi M., Schmidt-Erfurth U.M. (2014). Intraretinal cysts are the most relevant prognostic biomarker in neovascular age-related macular degeneration independent of the therapeutic strategy. Br. J. Ophthalmol..

[73] Elsharkawy M., Sharafeldeen A., Soliman A., Khalifa F., Widjajahakim R., Switala A., Elnakib A., Schaal S., Sandhu H.S., Seddon J.M. (2021). Automated diagnosis and grading of dry age-related macular degeneration using optical coherence tomography imaging. Investig. Ophthalmol. Vis. Sci..

[74] Elsharkawy M., Elrazzaz M., Ghazal M., Alhalabi M., Soliman A., Mahmoud A., El-Daydamony E., Atwan A., Thanos A., Sandhu H.S. (2021). Role of Optical Coherence Tomography Imaging in Predicting Progression of Age-Related Macular Disease: A Survey. Diagnostics.

[75] Gerendas B., Simader C., Deak G.G., Prager S.G.r., Lammer J., Waldstein S.M., Kundi M., Schmidt-Erfurth U. (2014). Morphological parameters relevant for visual and anatomic outcomes during anti-VEGF therapy of diabetic macular edema in the RESTORE trial. Investig. Ophthalmol. Vis. Sci..

[76] Welikala R.A., Fraz M.M., Dehmeshki J., Hoppe A., Tah V., Mann S., Williamson T.H., Barman S.A. (2015). Genetic algorithm based feature selection combined with dual classification for the automated detection of proliferative diabetic retinopathy. Comput. Med. Imaging Graph..

[77] Decencière E., Zhang X., Cazuguel G., Lay B., Cochener B., Trone C., Gain P., Ordonez R., Massin P., Erginay A. (2014). Feedback on a publicly distributed image database: The Messidor database. Image Anal. Stereol..

[78] Prasad D.K., Vibha L., Venugopal K. Early detection of diabetic retinopathy from digital retinal fundus images. Proceedings of the 2015 IEEE Recent Advances in Intelligent Computational Systems (RAICS).

[79] DIARETDB1—Standard Diabetic Retinopathy Database. http://www2.it.lut.fi/project/imageret/diaretdb1/index.html.

[80] Mahendran G., Dhanasekaran R. (2015). Investigation of the severity level of diabetic retinopathy using supervised classifier algorithms. Comput. Electr. Eng..

[81] Bhatkar A.P., Kharat G. Detection of diabetic retinopathy in retinal images using MLP classifier. Proceedings of the 2015 IEEE International Symposium on Nanoelectronic and Information Systems.

[82] Labhade J.D., Chouthmol L., Deshmukh S. Diabetic retinopathy detection using soft computing techniques. Proceedings of the 2016 International Conference on Automatic Control and Dynamic Optimization Techniques (ICACDOT).

[83] Rahim S.S., Palade V., Shuttleworth J., Jayne C. (2016). Automatic screening and classification of diabetic retinopathy and maculopathy using fuzzy image processing. Brain Inform..

[84] Bhatia K., Arora S., Tomar R. Diagnosis of diabetic retinopathy using machine learning classification algorithm. Proceedings of the 2016 2nd International Conference on Next Generation Computing Technologies (NGCT).

[85] Gulshan V., Peng L., Coram M., Stumpe M.C., Wu D., Narayanaswamy A., Venugopalan S., Widner K., Madams T., Cuadros J. (2016). Development and validation of a deep learning algorithm for detection of diabetic retinopathy in retinal fundus photographs. JAMA.

[86] Colas E., Besse A., Orgogozo A., Schmauch B., Meric N., Besse E. (2016). Deep learning approach for diabetic retinopathy screening. Acta Ophthalmol..

[87] Ghosh R., Ghosh K., Maitra S. Automatic detection and classification of diabetic retinopathy stages using CNN. Proceedings of the 2017 4th International Conference on Signal Processing and Integrated Networks (SPIN).

[88] Islam M., Dinh A.V., Wahid K.A. (2017). Automated diabetic retinopathy detection using bag of words approach. J. Biomed. Sci. Eng..

[89] Carrera E.V., González A., Carrera R. Automated detection of diabetic retinopathy using SVM. Proceedings of the 2017 IEEE XXIV International Conference on Electronics, Electrical Engineering and Computing (INTERCON).

[90] Somasundaram S.K., Alli P. (2017). A machine learning ensemble classifier for early prediction of diabetic retinopathy. J. Med. Syst..

[91] Kälviäinen R., Uusitalo H. (2007). DIARETDB1 diabetic retinopathy database and evaluation protocol. Medical Image Understanding and Analysis.

[92] ElTanboly A., Ismail M., Shalaby A., Switala A., El-Baz A., Schaal S., Gimel’farb G., El-Azab M. (2017). A computer-aided diagnostic system for detecting diabetic retinopathy in optical coherence tomography images. Med. Phys..

[93] Takahashi H., Tampo H., Arai Y., Inoue Y., Kawashima H. (2017). Applying artificial intelligence to disease staging: Deep learning for improved staging of diabetic retinopathy. PLoS ONE.

[94] Quellec G., Charrière K., Boudi Y., Cochener B., Lamard M. (2017). Deep image mining for diabetic retinopathy screening. Med. Image Anal..

[95] Ting D.S.W., Cheung C.Y.L., Lim G., Tan G.S.W., Quang N.D., Gan A., Hamzah H., Garcia-Franco R., San Yeo I.Y. (2017). Development and validation of a deep learning system for diabetic retinopathy and related eye diseases using retinal images from multiethnic populations with diabetes. JAMA.

[96] Wang Z., Yin Y., Shi J., Fang W., Li H., Wang X. Zoom-in-net: Deep mining lesions for diabetic retinopathy detection. Proceedings of the International Conference on Medical Image Computing and Computer-Assisted Intervention.

[97] Eladawi N., Elmogy M., Fraiwan L., Pichi F., Ghazal M., Aboelfetouh A., Riad A., Keynton R., Schaal S., El-Baz A. Early diagnosis of diabetic retinopathy in octa images based on local analysis of retinal blood vessels and foveal avascular zone. Proceedings of the 2018 24th International Conference on Pattern Recognition (ICPR).

[98] Dutta S., Manideep B., Basha S.M., Caytiles R.D., Iyengar N. (2018). Classification of diabetic retinopathy images by using deep learning models. Int. J. Grid Distrib. Comput..

[99] ElTanboly A., Ghazal M., Khalil A., Shalaby A., Mahmoud A., Switala A., El-Azab M., Schaal S., El-Baz A. An integrated framework for automatic clinical assessment of diabetic retinopathy grade using spectral domain OCT images. Proceedings of the 2018 IEEE 15th International Symposium on Biomedical Imaging (ISBI 2018).

[100] Zhang X., Zhang W., Fang M., Xue J., Wu L. Automatic classification of diabetic retinopathy based on convolutional neural networks. Proceedings of the 2018 International Conference on Image and Video Processing, and Artificial Intelligence. International Society for Optics and Photonics.

[101] Costa P., Galdran A., Smailagic A., Campilho A. (2018). A weakly-supervised framework for interpretable diabetic retinopathy detection on retinal images. IEEE Access.

[102] Pires R., Jelinek H.F., Wainer J., Goldenstein S., Valle E., Rocha A. (2013). Assessing the need for referral in automatic diabetic retinopathy detection. IEEE Trans. Biomed. Eng..

[103] Chakrabarty N. A deep learning method for the detection of diabetic retinopathy. Proceedings of the 2018 5th IEEE Uttar Pradesh Section International Conference on Electrical, Electronics and Computer Engineering (UPCON).

[104] Kwasigroch A., Jarzembinski B., Grochowski M. Deep CNN based decision support system for detection and assessing the stage of diabetic retinopathy. Proceedings of the 2018 International Interdisciplinary PhD Workshop (IIPhDW).

[105] EyePACS, LLC. http://www.eyepacs.com/.

[106] Li F., Liu Z., Chen H., Jiang M., Zhang X., Wu Z. (2019). Automatic detection of diabetic retinopathy in retinal fundus photographs based on deep learning algorithm. Transl. Vis. Sci. Technol..

[107] Nagasawa T., Tabuchi H., Masumoto H., Enno H., Niki M., Ohara Z., Yoshizumi Y., Ohsugi H., Mitamura Y. (2019). Accuracy of ultrawide-field fundus ophthalmoscopy-assisted deep learning for detecting treatment-naïve proliferative diabetic retinopathy. Int. Ophthalmol..

[108] Metan A.C., Lambert A., Pickering M. Small Scale Feature Propagation Using Deep Residual Learning for Diabetic Retinopathy Classification. Proceedings of the 2019 IEEE 4th International Conference on Image, Vision and Computing (ICIVC).

[109] Qummar S., Khan F.G., Shah S., Khan A., Shamshirband S., Rehman Z.U., Khan I.A., Jadoon W. (2019). A deep learning ensemble approach for diabetic retinopathy detection. IEEE Access.

[110] Sayres R., Taly A., Rahimy E., Blumer K., Coz D., Hammel N., Krause J., Narayanaswamy A., Rastegar Z., Wu D. (2019). Using a deep learning algorithm and integrated gradients explanation to assist grading for diabetic retinopathy. Ophthalmology.

[111] Sengupta S., Singh A., Zelek J., Lakshminarayanan V. (2019). Cross-domain diabetic retinopathy detection using deep learning. Appl. Mach. Learn. Int. Soc. Opt. Photonics.

[112] Hathwar S.B., Srinivasa G. Automated grading of diabetic retinopathy in retinal fundus images using deep learning. Proceedings of the 2019 IEEE International Conference on Signal and Image Processing Applications (ICSIPA).

[113] Li X., Shen L., Shen M., Tan F., Qiu C.S. (2019). Deep learning based early stage diabetic retinopathy detection using optical coherence tomography. Neurocomputing.

[114] Heisler M., Karst S., Lo J., Mammo Z., Yu T., Warner S., Maberley D., Beg M.F., Navajas E.V., Sarunic M.V. (2020). Ensemble deep learning for diabetic retinopathy detection using optical coherence tomography angiography. Transl. Vis. Sci. Technol..

[115] Alam M., Zhang Y., Lim J.I., Chan R.V., Yang M., Yao X. (2020). Quantitative optical coherence tomography angiography features for objective classification and staging of diabetic retinopathy. Retina.

[116] Zang P., Gao L., Hormel T.T., Wang J., You Q., Hwang T.S., Jia Y. (2020). DcardNet: Diabetic retinopathy classification at multiple levels based on structural and angiographic optical coherence tomography. IEEE Trans. Biomed. Eng..

[117] Ghazal M., Ali S.S., Mahmoud A.H., Shalaby A.M., El-Baz A. (2020). Accurate detection of non-proliferative diabetic retinopathy in optical coherence tomography images using convolutional neural networks. IEEE Access.

[118] Sandhu H.S., Elmogy M., Sharafeldeen A.T., Elsharkawy M., El-Adawy N., Eltanboly A., Shalaby A., Keynton R., El-Baz A. (2020). Automated diagnosis of diabetic retinopathy using clinical biomarkers, optical coherence tomography, and optical coherence tomography angiography. Am. J. Ophthalmol..

[119] Narayanan B.N., Hardie R.C., De Silva M.S., Kueterman N.K. (2020). Hybrid machine learning architecture for automated detection and grading of retinal images for diabetic retinopathy. J. Med. Imaging.

[120] Shankar K., Sait A.R.W., Gupta D., Lakshmanaprabu S., Khanna A., Pandey H.M. (2020). Automated detection and classification of fundus diabetic retinopathy images using synergic deep learning model. Pattern Recognit. Lett..

[121] Ryu G., Lee K., Park D., Park S.H., Sagong M. (2021). A deep learning model for identifying diabetic retinopathy using optical coherence tomography angiography. Sci. Rep..

[122] He A., Li T., Li N., Wang K., Fu H. (2021). CABNet: Category Attention Block for Imbalanced Diabetic Retinopathy Grading. IEEE Trans. Med. Imaging.

[123] Li T., Gao Y., Wang K., Guo S., Liu H., Kang H. (2019). Diagnostic assessment of deep learning algorithms for diabetic retinopathy screening. Inf. Sci..

[124] Saeed F., Hussain M., Aboalsamh H.A. (2021). Automatic diabetic retinopathy diagnosis using adaptive fine-tuned convolutional neural network. IEEE Access.

[125] Wang Y., Yu M., Hu B., Jin X., Li Y., Zhang X., Zhang Y., Gong D., Wu C., Zhang B. (2021). Deep learning-based detection and stage grading for optimising diagnosis of diabetic retinopathy. Diabetes/Metab. Res. Rev..

[126] Liu Z., Wang C., Cai X., Jiang H., Wang J. (2021). Discrimination of Diabetic Retinopathy From Optical Coherence Tomography Angiography Images Using Machine Learning Methods. IEEE Access.

[127] Sharafeldeen A., Elsharkawy M., Khalifa F., Soliman A., Ghazal M., AlHalabi M., Yaghi M., Alrahmawy M., Elmougy S., Sandhu H. (2021). Precise higher-order reflectivity and morphology models for early diagnosis of diabetic retinopathy using OCT images. Sci. Rep..

[128] Hsieh Y.T., Chuang L.M., Jiang Y.D., Chang T.J., Yang C.M., Yang C.H., Chan L.W., Kao T.Y., Chen T.C., Lin H.C. (2021). Application of deep learning image assessment software VeriSee™ for diabetic retinopathy screening. J. Formos. Med Assoc..

[129] Khan Z., Khan F.G., Khan A., Rehman Z.U., Shah S., Qummar S., Ali F., Pack S. (2021). Diabetic Retinopathy Detection Using VGG-NIN a Deep Learning Architecture. IEEE Access.

[130] Wang X., Han Y., Sun G., Yang F., Liu W., Luo J., Cao X., Yin P., Myers F.L., Zhou L. (2021). Detection of the Microvascular Changes of Diabetic Retinopathy Progression Using Optical Coherence Tomography Angiography. Transl. Vis. Sci. Technol..

[131] Abdelsalam M.M., Zahran M. (2021). A novel approach of diabetic retinopathy early detection based on multifractal geometry analysis for OCTA macular images using support vector machine. IEEE Access.

[132] Gao Z., Jin K., Yan Y., Liu X., Shi Y., Ge Y., Pan X., Lu Y., Wu J., Wang Y. (2022). End-to-end diabetic retinopathy grading based on fundus fluorescein angiography images using deep learning. Graefe’s Arch. Clin. Exp. Ophthalmol..

[133] Elsharkawy M., Sharafeldeen A., Soliman A., Khalifa F., Ghazal M., El-Daydamony E., Atwan A., Sandhu H.S., El-Baz A. (2022). A Novel Computer-Aided Diagnostic System for Early Detection of Diabetic Retinopathy Using 3D-OCT Higher-Order Spatial Appearance Model. Diagnostics.

[134] Zia F., Irum I., Qadri N.N., Nam Y., Khurshid K., Ali M., Ashraf I., Khan M.A. (2022). A Multilevel Deep Feature Selection Framework for Diabetic Retinopathy Image Classification. Comput. Mater. Contin.

[135] kag 2019 APTOS 2019 Blindness Detection. https://www.kaggle.com/c/aptos2019-blindness-detection..

[136] Tsai C.Y., Chen C.T., Chen G.A., Yeh C.F., Kuo C.T., Hsiao Y.C., Hu H.Y., Tsai I.L., Wang C.H., Chen J.R. (2022). Necessity of Local Modification for Deep Learning Algorithms to Predict Diabetic Retinopathy. Int. J. Environ. Res. Public Health.

[137] Das S., Saha S.K. (2022). Diabetic retinopathy detection and classification using CNN tuned by genetic algorithm. Multimed. Tools Appl..

[138] Singh H., Sharma V., Singh D. (2022). Comparative analysis of proficiencies of various textures and geometric features in breast mass classification using k-nearest neighbor. Vis. Comput. Ind. Biomed. Art.

[139] Sharafeldeen A., Elsharkawy M., Khaled R., Shaffie A., Khalifa F., Soliman A., Abdel Razek A.A., Hussein M.M., Taman S., Naglah A. (2021). Texture and shape analysis of diffusion-weighted imaging for thyroid nodules classification using machine learning. Med. Phys..

[140] Elsharkawy M., Sharafeldeen A., Taher F., Shalaby A., Soliman A., Mahmoud A., Ghazal M., Khalil A., Alghamdi N.S., Razek A.A.K.A. (2021). Early assessment of lung function in coronavirus patients using invariant markers from chest X-rays images. Sci. Rep..

[141] Zwanenburg A., Vallières M., Abdalah M.A., Aerts H.J.W.L., Andrearczyk V., Apte A., Ashrafinia S., Bakas S., Beukinga R.J., Boellaard R. (2020). The Image Biomarker Standardization Initiative: Standardized Quantitative Radiomics for High-Throughput Image-based Phenotyping. Radiology.

[142] Eltrass A.S., Salama M.S. (2020). Fully automated scheme for computer-aided detection and breast cancer diagnosis using digitised mammograms. IET Image Process..

[143] Salama M.S., Eltrass A.S., Elkamchouchi H.M. An Improved Approach for Computer-Aided Diagnosis of Breast Cancer in Digital Mammography. Proceedings of the 2018 IEEE International Symposium on Medical Measurements and Applications (MeMeA).

[144] Chetoui M., Akhloufi M.A., Kardouchi M. Diabetic Retinopathy Detection Using Machine Learning and Texture Features. Proceedings of the 2018 IEEE Canadian Conference on Electrical & Computer Engineering (CCECE).

[145] Nijalingappa P., Sandeep B. Machine learning approach for the identification of diabetes retinopathy and its stages. Proceedings of the 2015 International Conference on Applied and Theoretical Computing and Communication Technology (iCATccT).

[146] Radovic M., Ghalwash M., Filipovic N., Obradovic Z. (2017). Minimum redundancy maximum relevance feature selection approach for temporal gene expression data. BMC Bioinform..

[147] Jolliffe I.T. (2002). Principal Component Analysis.

[148] Akram M.U., Khalid S., Tariq A., Khan S.A., Azam F. (2014). Detection and classification of retinal lesions for grading of diabetic retinopathy. Comput. Biol. Med..

[149] Szymkowski M., Saeed E., Saeed K., Mariak Z. A simple algorithm for hard exudate detection in diabetic retinopathy using spectral-domain Optical Coherence Tomography. Proceedings of the Computer Graphics International Conference.

[150] Sleman A.A., Soliman A., Elsharkawy M., Giridharan G., Ghazal M., Sandhu H., Schaal S., Keynton R., Elmaghraby A., El-Baz A. (2021). A novel 3D segmentation approach for extracting retinal layers from optical coherence tomography images. Med. Phys..

[151] El-Baz A.S., Gimel’farb G.L., Suri J.S. (2016). Stochastic Modeling for Medical Image Analysis.

[152] Khansari M.M., Zhang J., Qiao Y., Gahm J.K., Sarabi M.S., Kashani A.H., Shi Y. (2019). Automated deformation-based analysis of 3D optical coherence tomography in diabetic retinopathy. IEEE Trans. Med. Imaging.

[153] Leela Jancy P., Lazha A., Prabha R., Sridevi S., Thenmozhi T. (2022). Hard Exudates Detection for Diabetic Retinopathy Early Diagnosis Using Deep Learning. Sustainable Communication Networks and Application.

[154] Holmberg O.G., Köhler N.D., Martins T., Siedlecki J., Herold T., Keidel L., Asani B., Schiefelbein J., Priglinger S., Kortuem K.U. (2020). Self-supervised retinal thickness prediction enables deep learning from unlabelled data to boost classification of diabetic retinopathy. Nat. Mach. Intell..

[155] Guo Y., Camino A., Wang J., Huang D., Hwang T.S., Jia Y. (2018). MEDnet, a neural network for automated detection of avascular area in OCT angiography. Biomed. Opt. Express.

[156] Hua C.H., Huynh-The T., Kim K., Yu S.Y., Le-Tien T., Park G.H., Bang J., Khan W.A., Bae S.H., Lee S. (2019). Bimodal learning via trilogy of skip-connection deep networks for diabetic retinopathy risk progression identification. Int. J. Med Inform..

[157] Gatys L.A., Ecker A.S., Bethge M. Image style transfer using convolutional neural networks. Proceedings of the IEEE Conference on Computer Vision and Pattern Recognition.

[158] Lakshminarayanan V., Kheradfallah H., Sarkar A., Jothi Balaji J. (2021). Automated Detection and Diagnosis of Diabetic Retinopathy: A Comprehensive Survey. J. Imaging.

